# Distributed Leadership Agency and Work Outcomes: Validation of the Italian DLA and Its Relations With Commitment, Trust, and Satisfaction

**DOI:** 10.3389/fpsyg.2020.00512

**Published:** 2020-03-31

**Authors:** Massimiliano Barattucci, Alessandro Lo Presti, Giambattista Bufalino, Thomas Jønsson, Manuel Teresi, Stefano Pagliaro

**Affiliations:** ^1^Faculty of Psychology, Università e-Campus, Novedrate, Italy; ^2^Department of Psychology, Università della Campania “Luigi Vanvitelli”, Caserta, Italy; ^3^Department of Educational Sciences, Università di Catania, Catania, Italy; ^4^Department of Psychology and Behavioral Sciences, Aarhus University, Aarhus, Denmark; ^5^Department of Neuroscience, Imaging and Clinical Sciences, Group Processes and Morality Lab (GPM-Lab), Università degli Studi “G. d’Annunzio” Chieti-Pescara, Chieti, Italy

**Keywords:** distributed leadership agency, validation, work outcomes, satisfaction, trust

## Abstract

Forms of collective leadership, such as distributed leadership, have become increasingly important. The need for measurement of the variables involved in the delegation processes represents a new challenge for organizations that want to ensure high-level working. The present study aimed to validate the Italian version of the distributed leadership agency (DLA). The study was carried out on 704 employees (doctors, nurses, clerks, staff workers, healthcare assistants, consultants, management) of an Italian public hospital, who were selected to complete a survey on organizational perceptions. Multiple confirmatory factor analyses (maximum likelihood) have been computed to explore the factorial structure of the DLA, along with associations with other work outcomes. Results about the Italian DLA confirmed the original trifactorial structure of the construct, suggested by Yukl (2002), through good fit indexes and reliability scores; moreover, consistent with the literature, DLA was strongly related to satisfaction, commitment, and trust. Results contribute to underline the robustness of the construct of DLA in different cultural sectors and provide a useful tool to be adopted in the Italian context.

## Introduction

The complex and continuous transformations involving organizations has progressively led, consistently with widespread managerialism, to an increasingly narrow focus on performance, on the standardization of production processes, and on the greater distribution of work and responsibilities (Clarke and Newman, 1997; Hardt and Negri, 2001). New operational and organizational scenarios are opened to which companies must cope in a timely and creative manner (Hall, 2002; Barattucci et al., 2018).

Work changes and leadership make no exception, becoming a dynamic and constantly evolving concept retaining primary importance from a strategic point of view (Teresi et al., 2019). Genuine leadership can no longer be limited to the mere proclamation of values to be followed, but it rather represents a shared function through which the leader can manage people and processes on a daily basis, through a distribution of power (Hall, 2002). As a result, recently, models and forms of collective leadership have gained increasing importance, and distributed leadership (DL), identifiable as a shared collective and widespread leadership practice, able to improve the capacity for corporate change, is definitely one worth to be mentioned. On the one hand, several scholars argued that DL encourages the horizontal development of power, redistributes responsibilities, and promotes capacity building (Harvey et al., 2003); on the other, despite the proliferation of definitions and approaches to DL, its measurement is not exempt from critical points and difficulties from several perspectives (Harris, 2008; Jønsson et al., 2016).

Consequently, the need for a valid, accurate, and reliable measurement of the variables involved in the delegation and empowerment processes represents a new challenge for organizations that want to ensure high-level working standards (Barattucci et al., 2017). In the present research, therefore, we aimed to validate the Italian version of the distributed leadership agency (DLA) scale and verify its applicability in different contexts.

### The Progressive Distribution of Leadership

Leadership, as a mean for managing company objectives, methods, and principles, has assumed a key role in corporate identity and performance processes (Kouzes and Posner, 1993). Scholars have focused on the processes of transmission of corporate values from the leader to the follower, as well as on the impact they have on other psychological and organizational variables (Graber and Kilpatrick, 2008; Mancheno et al., 2009). Other studies have examined, instead, styles and types of leadership and their effect on work experiences (Brown et al., 2005; Leung, 2008; Zehir et al., 2014; Dinc and Aydemir, 2014; Newman et al., 2017; Ning and Zhaoyi, 2017; Naeem et al., 2019).

In recent years, the concept of leadership has progressively lost its charm of romantic competence to be sought in the “strong man,” in favor of a progressive transition to a new era of studies on group management. Thus, a series of new conceptualizations strongly linked leadership to specific organizational situations [for instance, service leadership, transformational leadership, charismatic leadership, situational leadership, team leadership, etc. (Yukl et al., 2002; Jeppesen et al., 2011; Tian et al., 2016; Newman et al., 2017)]. Thus, the search for innate leadership skills to be emulated and taken as a reference by all the workers has been progressively overcome in favor of the identification of transversal skills, typical of the management, which can also be transferred to subordinate workers (Bolden, 2011; Corrigan, 2013).

### Implementing Leadership Distribution

During the last decades, the awareness about the importance of implementing a leadership distribution approach within organizations and, more generally, complex systems has grown (Gronn, 2003; Bush, 2014). Harris (2005) suggested three reasons explaining the widespread interest in DL: (1) its descriptive power that seems to capture the forms of implicit practice in professional learning communities and communities of practice; (2) its power of representation in inspiring alternative forms of organization to obsolete organizational structures that find it difficult to adapt to contemporary demands; (3) its regulatory power and the increase in what Gronn (2003) defined *greedy work* of leaders that needs to be actively shared.

Distributed leadership, in the form of collective skills, carefully constructed through professional collaboration, can positively influence work outcomes (Harris, 2013). Moreover, there is evidence of a positive relation between DL, organizational improvement, and innovation (Leithwood and Mascall, 2008; Harris, 2009; Heck and Hallinger, 2010). A large amount of empirical evidence about DL has been obtained by means of qualitative studies carried out primarily in educational institutions, even though some scholars pointed out that it can be profitably used and practiced in other contexts and organizations (mainly social and health sector; Chreim et al., 2010; Martin et al., 2015; Unterrainer et al., 2016; Unterrainer et al., 2017). Recently, several theoretical models that promote employee involvement in organizational leadership have been proposed around principles of organizational participation, shared leadership, and organizational democracy (Wegge et al., 2010).

### Measurement of DLA

The concept of DLA (Jønsson et al., 2016) has been advanced by combining the Activity Theory Approach (Spillane et al., 2001; Gronn, 2002, 2009; Spillane and Diamond, 2007) and the Cognitive Theory of Agency (Bandura, 1997), and referring to Yukl et al. (2002) metacategories of leadership behavior. Distributed leadership agency has been defined as “the degree to which workers experience being actively engaged in leadership activities within organizational change, managing tasks, and strengthening social relations at work” (Jønsson et al., 2016, pp. 910). This definition emphasizes the perspective of the worker as an agent: all organizational members, with or without formal leadership functions, can perform leadership activities. In other words, DLA represents a potential to empower employees, to share resources, and to actively participate in decision-making, not only delegating leadership tasks (Conger and Kanungo, 1988; Tian et al., 2016). Distributed leadership agency concerns general and concrete leadership tasks around three different categories of leadership behavior: *task-oriented*, *relationship-oriented*, and *change-oriented*. Starting from literature indications (Mayrowetz, 2008; Heck and Hallinger, 2010) and reviewing previous DL scales including agentic perspective (Hulpia and Devos, 2009), the DLA scale was developed to be applicable to various organizational settings and to overcome previous scales’ lack of theoretical validity (Jønsson et al., 2016). However, even if the original validation study highlighted that DLA captured workers’ active participation in leadership tasks, results did not support the three-factorial structure, because of the strong relationship between each other (Jønsson et al., 2016). Therefore, new studies are needed not only to examine this construct among other cultures but also to provide additional evidence about its nature and structure.

### The Present Research

Since in the Italian context there are no validated scales for the assessment of DL, the main aim of this study was to validate the Italian version of the DLA [13-item form by Jønsson et al. (2016)]^1^ and to measure the correspondence between the constructs under consideration and the questionnaire used. More specifically, the study aimed (a) to test the psychometric characteristics of the Italian DLA; (b) to test the factorial structure of the Italian DLA; (c) to test the reliability and the construct validity; (d) to confirm, basing on previous findings (Jønsson et al., 2016; Barattucci et al., unpublished), the relationships of DLA with different job outcomes (satisfaction, trust, commitment).

## Methods

### Translation Process

The translation process included all the 13 original items generated by the authors (Jønsson et al., 2016), following these phases: (1) two Italian work psychologists proficient in English translated the items individually, and then, a third researcher compared the translations and made a draft of the first agreed version; (2) the draft version was distributed for a pilot study to a small control sample to test for items readability (*N* = 43); (3) a back-translation of the draft version was performed by a native English speaker, with a comparison with the original English version; (4) the 13 items were presented to a sample of 21 workers with subsequent interviews in small groups (*N* = 4–6), to test the semantic congruence between the interpretation given by participants and the meaning of items in their original English version. In particular, item 13 of the Italian DLA seemed to be perceived somehow overlapping with item 12 and also not readily understood. Consequently, item 13 was excluded from the final version of the questionnaire.

### Participants and Procedure

The study took place from April to May 2019 and involved all the employees of an Italian public hospital, which were selected to complete a survey on organizational perceptions (*N* = 765). Questionnaires were distributed by trained researchers and, together with a research presentation, were presented to respondents in a paper-and-pencil format. After completion, the questionnaires were put in an anonymous envelope and returned collectively to the researcher. Completed paper questionnaires were collected after 2 weeks.

The total final sample included 704 employees (physicians, nurses, clerks, staff workers, healthcare assistants, consultants, management) (response rate, 92%). The sample was mostly made up of women [(*N* = 443 (63%); men: *N* = 261 (37%)], and the mean age was 45.73 (SD = 10.88) years; average organizational tenure was 7.31 (SD = 11.31) years, and general tenure was 19.22 (SD = 10.67) years. Healthcare assistants (19.9%) and nurses (18.1%) were the bigger subsamples; the rest of the sample was equally divided between clerks (15.1%), staff workers (14.8%), consultants (11.3%), doctors (11%), and managers (9.7%). Almost a third of the sample had a compulsory education [*N* = 153 (21.8%)] or a professional qualification [*N* = 62 (8.8%)], 26.9% of the workers had a high school degree (*N* = 189), and the remaining had a 3-year [*N* = 111 (15.9%)], a 5-year university degree [*N* = 124 (17.7%)], or a postdegree specialization [*N* = 62 (8.9%)].

### Measures

Participants filled in the following scales. The Italian version of the DLA (Jønsson et al., 2016) measures the degree to which organizational members experience being actively involved in leadership activities within organizational change, managing tasks, and strengthening social relations at work. The Italian DLA consists of 12 items on a 5-point Likert scale (0 = do not agree; 4 = totally agree) and three subscales made up of 2–3 items (task, change, relations).

*Affective commitment* was assessed through four items of the Italian form (Pierro et al., 1995) of the commitment scale by Meyer and Allen (1991) (e.g. “I would be very happy to spend the rest of my career with this organization”; α = 0.86; from 0 = “completely disagree” to 5 = “completely agree”).

*Perceived organizational trust* was measured with three items (e.g. “I believe that my company is fair”; from 1 = “completely disagree” to 7 = “completely agree”; α = 0.89), derived and adapted from the international literature (Colquitt and Rodell, 2015).

*Job satisfaction* was measured with three items (“How satisfied are you with…?”; α = 0.71) translated from the *overall job satisfaction* (Cammann et al., 1983), and concerning different aspects of the work experience (professional involvement, work environment, and career), items were assessed on a five-point Likert-scale, ranging from 0 = “totally dissatisfied” to 4 = “totally satisfied.”

### Data Analysis

Data have been processed with SPSS version 21 (IBM, Chicago, IL) and Lisrel version 9.30 (Scientific Software International Inc., Skokie, IL). Missing values have been replaced with the expected maximization method (EM method). Multivariate outliers have been removed after the calculation of Mahalanobis distances. Items’ distributions have been checked computing asymmetry and kurtosis indexes.

Multiple confirmatory factor analyses (CFAs; estimation method: maximum likelihood) have been computed to explore the factorial structure of the distributive leadership scale, along with associations with other study variables. To estimate model fit and compare competing measurement models, we relied on several goodness-of-fit indexes that minimized the likelihood of types I and II errors (Hu and Bentler, 1999): the χ^2^, comparative fit index (CFI), non-normed fit index (NNFI), standardized root mean residual (SRMR), and root mean square error of approximation (RMSEA). A significant χ^2^ can indicate a poorly fitting model, but being this test affected by sample size, it is not reliable in larger ones. Criteria for the goodness-of-fit indices can range from less (CFI, NNFI ≥ 0.90; SRMR, RMSEA ≤ 0.10) to more conservative (CFI, NNFI ≥ 0.95; SRMR, RMSEA ≤ 0.08; Hu and Bentler, 1999), but models’ goodness-of-fit evaluation should include evidence from all sources for subsequent acceptance or rejection.

McDonald ω and Cronbach α’s were computed for verifying the scales internal consistency.

## Results

Only seven missing values (over 12,672 expected cells) have been identified and replaced through the EM method. Subsequently, through the computation of Mahalanobis distances, 25 multivariate outliers have been identified and thus removed. The final sample then consisted of 679 cases. Subsequently, the first set of analyses was focused on verifying the factorial structure of the distributive leadership scale, contrasting alternative models. Asymmetry and kurtosis indexes have been computed for all 12 items of the distributive leadership scale. Asymmetry values ranged between -0.52 and -0.00, whereas kurtosis values were between -1.22 and -0.48, thus showing that assumptions of normality were not violated [i.e. values were below the ±1.96 cutoff as recommended by Schaufeli et al. (2006)].

Six different competing measurement models were estimated (Table 1). The first model (model 1) to be estimated consisted of all 12 items loading on a single factor. Goodness-of-fit indexes were not satisfactory [χ^2^ = 795.85, degrees of freedom (df) = 54, RMSEA = 0.142, SRMR = 0.0516, CFI = 0.885, NNFI = 0.859]. A second model (model 2) has been estimated with the 12 items loading on three different factors, as theorized by Jønsson et al. (2016). A remarkable improvement in goodness-of-fit indexes than model 1 could be observed, although these indexes were still not adequate (χ^2^ = 628.27, df = 51, RMSEA = 0.129, SRMR = 0.0541, CFI = 0.910, NNFI = 0.884). Finally, a third model with only seven items loading on a single factor and consistent with the one validated by Jønsson et al. (2016) was tested. Even in this case, goodness-of-fit indexes’ values were not satisfactory (χ^2^ = 249.25, df = 14, RMSEA = 0.157, SRMR = 0.0619, CFI = 0.929, NNFI = 0.893).

**TABLE 1:** Measurement models comparison.

	χ^2^	df	RMSEA	SRMR	CFI	NNFI
Model 1: One factor (12 items)	795.85	54	0.142	0.0516	0.885	0.859
Model 2: Three-factor model (12 items)	628.27	51	0.129	0.0541	0.910	0.884
Model 3: One factor (7 items)	249.25	14	0.157	0.0619	0.929	0.893
Model 4: Second-order three-factor model (8 items)	81.30	17	0.075	0.0207	0.984	0.974
Model 5: One factor (8 items)	284.38	20	0.140	0.040	0.935	0.909

A closer inspection of the first three measurement models, and in particular of models 2 and 3, suggested that a more explorative approach should be adopted and that a three-factor structure could be a more viable option to explore further. In particular, the examination of modification indexes and items’ cross-loadings suggested that items 2, 6, 8, and 12 cross-loaded on factors other than their original one and then that their removal could improve the measurement model fit. After removing these four items, a fourth model (model 4) has been estimated with three distinct factors loading on a common second-order factor, showing good goodness-of-fit indexes (χ^2^ = 81.30, df = 17, RMSEA = 0.075, SRMR = 0.0207, CFI = 0.984, NNFI = 0.974). For the sake of completeness, an additional measurement model was estimated, including the final eight retained items loading on a single factor. The significant worsening of goodness-of-fit indexes (χ^2^ = 284.38, df = 20, RMSEA = 0.140, SRMR = 0.040, CFI = 0.935, NNFI = 0.909) provided additional evidence that the second-order three-factor solution was a more adequate and viable structure. In summary, items 1 and 4 loaded on the first factor (change); items 3, 5, and 7 loaded on the second factor (task), whereas items 9, 10, and 11 on the third factor (relation).

McDonald ω for the whole scale was equal to 0.93, whereas Cronbach α was 0.84 for the first factor (change), 0.81 for the second one (task), and 0.92 for the last one (relation).

**FIGURE 1:**
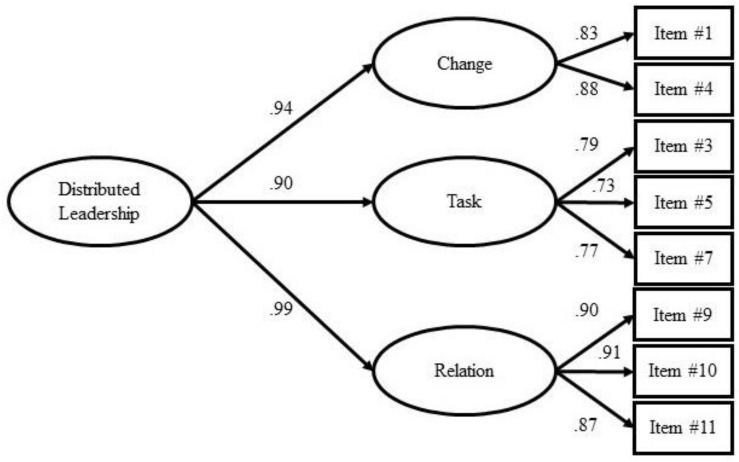
Definitive factorial model.

Item loadings ranged between 0.73 (item 5) and 0.91 (item 10). Factor loadings on the second-order factor were 0.94 (change), 0.90 (task), and 0.99 (relation), respectively. Figure 1 depicts the final factorial solution.

Before testing the correlations between study variables, sampling adequacy was computed through the Kaiser-Meyer-Olkin, returning a satisfactory value of 0.90. Asymmetry and kurtosis indexes have been computed for the items of the other three measures (i.e. affective commitment, trust, job satisfaction). Asymmetry values ranged between -0.58 and -0.06, whereas kurtosis values between -0.96 and -0.26, thus showing that assumptions of normality were not violated.

A measurement model including the distributive leadership second-order three-factor structure, as emerged previously along with affective commitment, perceived organizational trust, and job satisfaction, was developed. Factors were allowed to correlate with each other (standardized scores were used). The model showed good goodness-of-fit indexes (χ^2^ = 340.82, df = 126, RMSEA = 0.050, SRMR = 0.0366, CFI = 0.971, NNFI = 0.965). Distributive leadership positively correlated with trust (*r* = 0.22, *p* < 0.001), affective commitment (*r* = 0.28, *p* < 0.001), and job satisfaction (*r* = 0.27, *p* < 0.001).

Table 2 depicts the zero-order correlation between study variables along with descriptive statistics and Cronbach α’s.

**TABLE 2:** Zero-order correlations between study variables.

	Mean (SD)	1	2	3	4
(1) Distributive leadership	3.25 (1.03)	(0.93)			
(2) Trust	4.92 (1.47)	0.21***	(0.91)		
(3) Affective commitment	3.51 (1.11)	0.25***	0.47***	(0.82)	
(4) Job satisfaction	2.76 (0.83)	0.21***	0.45***	0.61***	(0.70)

***p < 0.001. Cronbach α’s on the diagonal.

## Discussion

The present study aimed to contribute to the validation of the Italian version of the DLA scale in the healthcare sector. Results clarified that the Italian DLA respects the trifactorial structure of the construct with good fit indexes and reliability; moreover, correlation analyses confirmed previous literature findings highlighting a strong relation between DLA and other delegation processes with important work outcomes (Jønsson et al., 2016; Unterrainer et al., 2017).

Thus, the three-factorial structure of the Italian DLA (task, change, relations) was empirically supported; the best definitive factorial solution (three factors) for the Italian version of the DLA scale was composed of eight items (two items for Task dimension, three items for change and relations dimension). The Italian eight-item DLA showed a very good global reliability and high internal consistency of all three subscales. Distributed leadership agency measures workers’ spontaneous contribution in leadership tasks and exhibited a strong positive relationship with important work and organizational outcomes (satisfaction, trust, and commitment; Jønsson et al., 2016; Unterrainer et al., 2017). Overall, results contribute to underline the robustness of the construct of DLA in different cultural contexts, adding to the generalizability of research in DL to various sectors.

### Limitations and Further Research

Despite the construct’s popularity, scholars have not reached a unanimous definition of DL (Bennett et al., 2003; Lakomski, 2008; Mayrowetz, 2008), and some argue that, because of its multidimensional nature, prior formulations have been used vaguely (Hartley, 2007; Torrance, 2009) or were rather uncritical (Young, 2009; Jones, 2014). Distributed leadership remains, therefore, a disputed topic (Grint, 2005), a free-floating concept (Young, 2014), and “a way of thinking about leadership.” In this scenario, a unanimously agreed-upon measurement of such a dynamic construct can be difficult.

A limitation of the present research, moreover, could concern the sample: participants were recruited in the health sector, a choice that could partly limit its results generalizability; in the future, it will certainly be necessary to evaluate the use of DLA in other work contexts and sectors.

In this study, we examined the factorial solution of the distributive leadership scale recurring to structural equation modeling, which is a statistical approach widely used in social sciences (Sheng, 2017; Kwok et al., 2018). However, future studies could adopt alternative statistical approaches such as partial least squares (PLS), which is attracting increasing scholarly attention (Hair and Sarstedt, 2019), for instance, for the sake of comparing results obtained with different statistical approaches (e.g. SEM vs. PLS).

### Practical Implications

The significant positive relationship between DL, different work outcomes, and leadership perceptions (transformational and empowering leadership) stresses how important it is for leaders to develop a culture in which all the actors of the system are eager to participate in leadership activities, but also recognized and appreciated for the effective assumption of responsibility.

Distributed leadership cannot be traced back to a mere distribution of tasks – which moreover refers to a functionalistic logic of school organization management – but in reality requires a change of mindset and a “letting go” (Duignan, 2006, p.107), especially for those leaders who are used to centralizing and leading in an autocratic manner. The quality of these relationships affects every choice and every event that occurs within organizations, including the quality and impact of leadership itself (Duignan and Bhindi, 1997).

In order to stimulate positive outcomes and innovative behavior among workers in organizations, managers may benefit from training in DL strategies, to better manage groups with common objectives and goals, add roles and responsibilities, empower assistant leaders, design group model with clear role definitions, increase the amount of assistant leaders, and coach and facilitate teams to work in a democratic way (Duignan, 2006; Eberly et al., 2013; Bouwmans et al., 2019). Moreover, the results may point toward Human resource management initiatives that strengthen workers’ self-efficacy, such as competence development.

### Closing Remarks

The idea of DL cannot be considered simply as a substitute for individual leadership, but rather as an essential complement that facilitates and encompasses both individual and collective dimensions. Distributed leadership is neither guarantee of better performance, nor a panacea for success; on the contrary, much depends on how the leadership is distributed and the intentions that are at the base of an active involvement of the workers. If “delegation is a mode of transaction in which the leader refers to a subordinate what to do” (Lowham, 2007, p. 71), in the DL process “the actors synchronize the actions undertaken taking into consideration their own plans, those of their peers, and their sense of unit membership” (Gronn, 2002, p. 431).

Distributed leadership is the result of continuous processes of interactions and negotiations between all members of the organization, because the latter contributes to build and rebuild the working reality in a productive and collaborative manner (Gronn, 2002; Lowham, 2007).

Sharing leadership calls into question a rethinking of the way in which all stakeholders in a business community reconsider the identity traits of leadership itself, and in particular its founding assumptions: concepts such as those of power, authority, influence, position, role, responsibility, and accountability, as well as personal and professional relationships, must be revisited and revised if necessary. The idea of a hybrid configuration of leadership that integrates individual, collective, and situational dimensions in the practice of leadership allows us to indicate when and why particular configurations are more effective and/or desirable than others (Bolden, 2011).

## Data Availability Statement

All datasets generated for this study are included in the article/supplementary material.

## Ethics Statement

The studies involving human participants were reviewed and approved by the Ethical Committee of the E-Campus University. The patients/participants provided their written informed consent to participate in this study.

## Author Contributions

MB, GB, and TJ contributed to the conception and design of the study. GB and MB organized the database. AL performed the statistical analysis. MB wrote the first draft of the manuscript. AL, SP, MT, GB, and MB wrote the sections of the manuscript. All authors contributed to the manuscript revision, read and approved the submitted version.

## Conflict of Interest

The authors declare that the research was conducted in the absence of any commercial or financial relationships that could be construed as a potential conflict of interest.

## Footnotes

1 Items reported in Jønsson, Unterrainer, and Kähler (2020). The Role of Distributed Leadership Agency in Participatory Employee Innovation. Manuscript under review.

## References

[B1] Bandura A. (1997). The anatomy of stages of change. *Am. Health Promot.* 12 8–10. 10.4278/0890-1171-12.1.8 10170438

[B2] Barattucci M. Alfano V. Amodio S. (2017). The company judged from the inside: diversification, equity and justice in organizations. *J. Psychol. Educ. Res.* 25 65–81.

[B3] Barattucci M. Cafagna D. Bocciolesi E. Fraschetti V. (2018). Active training techniques for outplacement: does group training improve placement? *Encyclopaideia* 22 1–10. 10.6092/issn.1825-8670/8422

[B4] Bennett N. Wise C. Woods P. Harvey J. (2003). *Distributed Leadership: Full Report.* Nottingham: National College for School Leadership.

[B5] Bolden R. (2011). Distributed leadership in organizations: a review of theory and research. *Intern. J. Manag. Rev.* 13 251–269. 10.1111/j.1468-2370.2011.00306.x

[B6] Bouwmans M. Runhaar P. Wesselink R. Mulder M. (2019). Towards distributed leadership in vocational education and training schools: the interplay between formal leaders and team members. *Educ. Manag. Admin. Leadersh.* 47 555–571. 10.1177/1741143217745877

[B7] Brown M. E. Treviño L. K. Harrison D. A. (2005). Ethical leadership: a social learning per-spective for construct development and testing. *Organ. Behav. Hum. Deci. Process.* 97 117–134. 10.1016/j.obhdp.2005.03.002

[B8] Bush T. (2014). Applying distributed leadership across contexts. *Educ. Manag. Admin. Leadersh.* 42 601–602. 10.1177/1741143214541369

[B9] Cammann C. Fichman M. Jenkins G. D. Klesh J. R. (1983). “Assessing the attitudes and perceptions of organizational members,” in *Taking the Measure Of Work. A Guide To Validated Scales For Organizational Research And Diagnosis*, ed. Fields D. L., (Thousand Oaks, CA: Sage), 1121–1141.

[B10] Chreim S. Williams B. E. Janz L. Dastmalchian A. (2010). Change agency in a primary health care context: the case of distributed leadership. *Health Care Manage. Rev.* 35 187–199. 10.1097/HMR.0b013e3181c8b1f8 20234224

[B11] Clarke J. H. Newman J. (1997). *The Managerial State: Power, Politics and Ideology in the Remaking of Social Welfare.* London: Sage.

[B12] Colquitt J. A. Rodell J. B. (2015). “Measuring justice and fairness,” in *Oxford Library of Psychology. The Oxford Handbook Of Justice In The Workplace*, eds Cropanzano R. S. Ambrose M. L., (New York, NY: Oxford University Press), 187–202. 10.1093/oxfordhb/9780199981410.013.8

[B13] Conger J. A. Kanungo R. N. (1988). The empowerment process: integrating theory and practice. *Acad. Manag. Rev.* 13 471–482. 10.2307/258093

[B14] Corrigan J. (2013). Distributed leadership: rhetoric or reality? *J. Higher Educ. Policy Manag.* 35 66–71. 10.1080/1360080X.2013.748479

[B15] Dinc M. S. Aydemir M. (2014). Ethical leadership and employee behaviours: an empirical study of mediating factors. *Intern. J. Bus. Govern. Ethic.* 9 293–312. 10.1504/IJBGE.2014.06473

[B16] Duignan P. (2006). *Educational Leadership: Key Challenges And Ethical Tensions.* New York, NY: Cambridge University Press.

[B17] Duignan P. A. Bhindi N. L. (1997). Authenticity in Leadership. *J. Educ. Admin.* 35 195–210. 10.1108/09578239710170119

[B18] Eberly M. B. Johnson M. D. Hernandez M. Avolio B. J. (2013). An integrative process model of leadership: examining loci, mechanisms, and event cycles. *Am. Psychol.* 68 427–443. 10.1037/a0032244 23528243

[B19] Graber D. R. Kilpatrick A. O. (2008). Establishing values-based leadership and value systems in healthcare organizations. *J. Health Hum. Serv. Admin.* 31 179–197. 18998522

[B20] Grint K. (2005). *Leadership: Limits And Possibilities.* Basingstoke: Palgrave Macmillan.

[B21] Gronn P. (2002). Distributed leadership as a unit of analysis. *Leadersh. Q.* 13 423–451. 10.1016/S1048-9843(02)00120-120

[B22] Gronn P. (2003). *The New Work of Educational Leadership: Changing Leadership Pratices in An Era Of School Reform.* London: Paul Chapman.

[B23] Gronn P. (2009). Leadership configurations. *Leadership* 5 381–394. 10.1177/1742715009337770

[B24] Hair J. F. Jr. Sarstedt M. (2019). Factors versus composites: guidelines for choosing the right structural equation modeling method. *Project Manag. J.* 50 619–624. 10.1177/8756972819882132

[B25] Hall D. T. (2002). *Careers In And Out Of Organizations.* Thousand Oaks, CA: Sage.

[B26] Hardt M. Negri A. (2001). *Empire.* Cambridge: Harvard University Press.

[B27] Harris A. (2005). Reflection on distributed leadership. *Manag. Educ.* 19 10–12. 10.1177/08920206050190020301

[B28] Harris A. (2008). Distributed leadership: according to evidence. *J. Educ. Admin.* 46 172–188. 10.1108/09578230810863253

[B29] Harris A. (2009). *Distributed Leadership: Different Perspectives.* London: Springer.

[B30] Harris A. (2013). Distributed leadership: friend or foe? *Educ. Manag. Admin. Leadersh.* 41 545–554. 10.1177/1741143213497635

[B31] Hartley D. (2007). The emergence of distributed leadership in education: why now? *Br. J. Educ. Stud.* 55 202–214. 10.1111/j.1467-8527.2007.00371.x

[B32] Harvey S. Royal M. Stout D. (2003). Instructor’s trasformational leadership: university student attitudes and ratings. *Psychol. Rep.* 92 395–402. 10.2466/pr0.2003.92.2.395 12785619

[B33] Heck R. H. Hallinger P. (2010). Testing a longitudinal model of distributed leadership effects on school improvement. *Leadersh. Q.* 21 867–885. 10.1016/j.leaqua.2010.07.013

[B34] Hu L. T. Bentler P. M. (1999). Cutoff criteria for fit indexes in covariance structure analysis: Conventional criteria versus new alternatives. *Struct. Equ. Model.* 6 1–55. 10.1080/10705519909540118

[B35] Hulpia H. Devos G. (2009). Exploring the link between distributed leadership and job satisfaction of school leaders. *Educ. Stud.* 35 153–171. 10.1080/03055690802648739

[B36] Jeppesen H. J. Jønsson T. S. Shevil M. (2011). Employee attitudes to the distribution of organiza-tional influence: Who should have the most influence on which issues? *Econ. Indus. Trial Democr.* 32 69–86. 10.1177/0143831X10372432

[B37] Jones S. (2014). Distributed leadership: a critical analysis. *Leadership* 10 129–141. 10.1177/1742715011433525

[B38] Jønsson T. Unterrainer C. Jeppesen H.-J. Jain H. K. (2016). Measuring distributed leadership agency in a hospital context: development and validation of a new scale. *J. Health Organ. Manag.* 30 908–926. 10.1108/JHOM-05-2015-2068 27681024

[B39] Kouzes J. M. Posner B. Z. (1993). *Leadership Practices Inventory. A Self-Assessment and Anal-ysis, Expanded.* San Francisco, CA: Jossey-Bass.

[B40] Kwok O. M. Cheung M. W. Jak S. Ryu E. Wu J. Y. (2018). Recent advancements in structural equation modeling (sem): from both methodological and application perspectives. *Front. Psychol.* 9:1936. 10.3389/fpsyg.2018.01936 30356842 PMC6190731

[B41] Lakomski G. (2008). Functionally adequate but casually idle: w(h)ither distributed leadership. *J. Educ. Admin.* 46 159–171. 10.1108/09578230810863244

[B42] Leithwood K. Mascall B. (2008). Collective leadership effects on student achievement. *Educ. Admin. Q.* 44 529–561. 10.1177/0013161X08321221

[B43] Leung K. (2008). Matching ethical work climate to in-role and extra-role behaviors in a collectivist work setting. *J. Bus. Ethic.* 79 43–55. 10.1007/s10551-007-9392-9396

[B44] Lowham E. (2007). *Too Many Cooks? Distributed Leadership in State Brownfields Remediation and Redevelopment Programs.* Ph. D. thesis, University of Colorado, Boulder, CO.

[B45] Mancheno S. L. Endres G. M. Polak R. Athanasaw Y. (2009). The individual cultural val-ues and job satisfaction of the transformati-onal leader. *Organ. Dev. J.* 27 9–21.

[B46] Martin G. Beech N. MacIntosh R. Bushfield S. (2015). Potential challenges facing distributed leadership in health care: evidence from the UK National Health Service. *Sociol. Health Illness* 37 14–29. 10.1111/1467-9566.12171 25529349

[B47] Mayrowetz D. (2008). Making sense of distributed leadership: exploring the multiple usages of the concept in the field. *Educ. Admin. Q.* 44 424–435. 10.1177/0013161X07309480

[B48] Meyer J. P. Allen N. J. (1991). A three-component conceptualization of organizational commit-ment. *Hum. Resour. Manag. Rev.* 1 61–98. 10.1016/1053-4822(91)90011Z

[B49] Naeem R. M. Weng Q. D. Hameed Z. Rasheed M. I. (2019). Ethical leadership and work engagement: a moderated mediation model. *Ethic. Behav.* 30 63–82. 10.1080/10508422.2019.1604232

[B50] Newman A. Round H. Bhattacharya S. Roy A. (2017). Ethical climates in organizations: a review and research agenda. *Bus. Ethic. Q.* 27 475–512. 10.1017/beq.2017.23

[B51] Ning N. Zhaoyi L. (2017). Psychological contract breach, organizational disidentification, and employees’ unethical behavior: organizational ethical climate as moderator. *Soc. Behav. Pers. Intern. J.* 45 1409–1424. 10.2224/sbp.6708

[B52] Pierro A. Lombardo I. Fabbri S. Di Spirito A. (1995). Evidenza empirica della validità discriminante delle misure di Job Involvement e organizational commitment. *Test. Psicometr. Metodol.* 2 5–18.

[B53] Schaufeli W. B. Bakker A. B. Salanova M. (2006). The measurement of work engagement with a short questionnaire a cross-national study. *Educ. Psychol. Measur.* 66 701–716. 10.1177/0013164405282471

[B54] Sheng Y. (2017). Fitting psychometric models: issues and new developments. *Front. Psychol.* 8:856. 10.3389/fpsyg.2017.00856 28611708 PMC5447066

[B55] Spillane J. Diamond J. (2007). *Distributed Leadership in Practice.* New York, NY: Teachers College Press.

[B56] Spillane J. P. Halverson R. Diamond J. B. (2001). Investigating school leadership practice: a distributed perspective. *Educ. Res.* 30 23–28. 10.3102/0013189X030003023

[B57] Teresi M. Pietroni D. D. Barattucci M. Giannella V. A. Pagliaro S. (2019). Ethical climate(s), organizational identification, and employees’ behavior. *Front. Psychol.* 10:1356. 10.3389/fpsyg.2019.01356 31275196 PMC6593040

[B58] Tian M. Risku M. Collin K. (2016). A meta-analysis of distributed leadership from 2002 to 2013: theory development, empirical evidence and future research focus. *Educ. Manag. Admin. Leadersh.* 44 146–164. 10.1177/1741143214558576

[B59] Torrance M. (2009). The rise of a global infrastructure market through relational investing. *Econ. Geogr.* 85 75–97. 10.1111/j.1944-8287.2008.01004.x

[B60] Unterrainer C. Jeppesen H. Jønsson T. Weber W. (2016). Improving employees innovative behavior at work: the impact of organizational participation in decision-making, distributed leadership agency and employees occupational self-efficacy: OR1438. *Intern. J. Psychol.* 51:780.

[B61] Unterrainer C. Jeppesen H. J. Jønsson T. (2017). Distributed leadership agency and its relationship to individual autonomy and occupational self-efficacy: a two wave-mediation study in denmark. *Hum. Manag. J.* 2 57–81. 10.1007/s41463-017-0023-9

[B62] Wegge J. Jeppesen H. J. Weber W. G. Peace C. L. Silva S. A. Pundt A. (2010). Promoting work motivation in organizations: should employee involvement in organizational leadership become a new tool in the organizational psychologist’s kit? *J. Pers. Psychol.* 9 154–171. 10.1027/1866-5888/a000025

[B63] Young H. (2009). Un Critical times? Situating distributed leadership in the field. *J. Educ. Admin. Hist.* 41 377–389. 10.1080/00220620903211588

[B64] Young H. (2014). Moving beyond distributed leadership to distributed forms: a contextual and socio-cultural analysis of two New Zealand secondary schools. *Lead. Manag.* 20 88–103.

[B65] Yukl G. A. (2002). *Leadership in Organizations*, 5th Edn Upper Saddle River: Prentice Hall.

[B66] Yukl G. Gordon A. Taber T. (2002). A hierarchical taxonomy of leadership behavior: integrat-ing a half century of behavior research. *J. Leadersh. Organ. Stud.* 9 15–32. 10.1177/107179190200900102

[B67] Zehir C. Müceldili B. Altındağ E. Sehitoğlu Y. Zehir S. (2014). Charismatic leadership and organizational citizenship behavior: the mediating role of ethical climate. *Soc. Behav. Pers. Intern. J.* 42 1365–1376. 10.2224/sbp.2014.42.8.1365

